# IMPORTANCE OF ENGAGING IN DIALOGUE WITH THE POPULATION AFTER A NUCLEAR ACCIDENT

**DOI:** 10.1093/rpd/ncw320

**Published:** 2016-11-24

**Authors:** Hans Vanmarcke

**Affiliations:** 1 Belgian Nuclear Research Centre SCK•CEN, Boeretang 200, Mol 2400, Belgium

## Abstract

Human behaviour is primarily driven by perceptions and this is particularly important in the aftermath of a nuclear accident. One of the main lessons we can draw from the Chernobyl and Fukushima accidents is that once the acute phase of the accident is over, it is important to engage in dialogue with the affected population. Science-based government measures, imposed from above, give rise to much opposition. Examples of this are the aversion of having to live in a contaminated territory, the reluctance of consumers to buy even slightly contaminated food and the opposition of most evacuees to return to their old homes. The continuing controversy within the scientific community about low-dose risks, which results in conflicting messages to the population, is also not very helpful. A way to deal with these problems is by empowering the affected population by establishing a kind of formal consultation structure funded by the authorities but operated by the local community. This will give the population the feeling of having some control over the situation. Such a participatory approach is very demanding for the authorities, but is likely to change the state of mind of the affected people from victims to survivors.

## INTRODUCTION

The Chernobyl accident 30 years ago and the Fukushima accident 5 years ago are good illustrations of the inability of society and the authorities in particular to effectively deal with the disruptive impact of the countermeasures on the well-being of the affected population. And in particular to deal with the concern and anxiety of the evacuated people close to the accident site, most of whom have still not returned home, and the people further away who first had to stay inside their homes for a period of time and now have to live their lives in a contaminated environment. A lot of these people have difficulties to resume to a normal life as they still define themselves as victims of the accident, even if from a scientific point of view their risks from the exposure at the time of the accident, or from the remaining environmental contamination are small compared to the risks of everyday life. The controversy in the scientific community on the significance and magnitude of the health effects of low doses of ionizing radiation, where on the one hand some are convinced of supra-linear detrimental effects, while others claim that doses just above the natural background are beneficial, is not helpful and confuses the affected populations still struggling with the considerable impact of the accident on their daily life. A difference in mind-set between the techno-scientific community and the affected population is at the base of this lack of understanding. While experts tend to focus on the facts, the population is mainly driven by their perception of the risk, which is subjective in nature and closely related to how they think and feel about nuclear energy and radiation risks in general. This attitude is not something specific for nuclear accidents but of a general nature as expressed by Renn in 2008^(1)^ ‘Human behaviour is primarily driven by perceptions and not by facts’. Hence, a successful communication has to address the patterns and rationale of risk perception in general and the perception of ionizing radiation in particular. The way the affected population perceives the risk is influenced by a lot of factors, like the involuntary nature of the exposure, the involvement of women and children, the unfamiliarity with the risk, etc. Radiation risk perception is a well-studied subject. More information on the consistent patterns and rationale of risk perception can be found at^(2–5)^.

## EXAMPLES OF HEIGHTENED SOCIETAL CONCERN

If we accept that human behaviour is primarily driven by perceptions and not by facts then the central question is how to change the negative perception of the affected population after a nuclear accident into a more positive perception, or in other words how to change their state of mind from victims to survivors? A few examples from the Chernobyl and Fukushima accidents to illustrate the problems of our current approach.

### A strong aversion to live in a contaminated territory

Large areas in Belarus, Ukraine and Russia suffered high levels of radioactive contamination due to the Chernobyl accident. ^137^Cs is the most important radionuclide in the long term. In total, an area of ~150 000 km^2^ (five times the surface area of Belgium) was contaminated with more than 37 kBq/m² ^137^Cs and 3100 km², mainly in Belarus, with more than 1480 kBq/m² (Table 1)^(6)^. The contamination of the soil is not homogeneous at all due to the variable weather conditions (wind direction, precipitation, etc.) during the atmospheric releases. Several studies have demonstrated that people living in the most seriously affected regions in Belarus, Ukraine and Russia show higher rates of health complaints and psychological distress than inhabitants of unexposed regions^(3)^.

**Table 1.** Surface area contaminated with ^137^Cs (in kBq/m²) in the three most affected republics of the former Soviet Union^(6)^.

Country	37–185	185–555	555–1480	>1480	Total (≥37)
Belarus (km²)	30 000	10 000	4200	2200	46 400
Russia (km²)	50 000	5500	2100	300	57 900
Ukraine (km²)	37 000	3200	900	600	41 700
Total (km²)	117 000	18 700	7200	3100	146 000

For comparison, the soil contamination in Belgium with ^137^Cs was a few kBq/m² at the time of the Chernobyl accident, in about equal measure from Chernobyl fallout and from atmospheric nuclear weapons testing in the late 1950s and early 1960s.

UNSCEAR's estimation of the total effective dose accumulated during the first 10 years by the 5 million people living in the most contaminated areas is not so high, despite the high contamination level of the soil:

- 0–5 mSv:59% (less than the annual average exposure in Belgium: 4.6 mSv^(7–8)^)
- 5–10 mSv:20% (about one CT-scan)
- 10–20 mSv:13%
- 20–50 mSv:6.9% (more than the annual limit for radiation workers: 20 mSv)
- 50–100 mSv:0.9%
- 100–200 mSv:0.02 %
- >200 mSv:0.002%

These values do not take the thyroid doses from the short lived iodine isotopes into account, which dominated the exposure during the first few months.

The additional exposure due to the Chernobyl accident of the people living in the most contaminated areas is in the range of exposures from natural radiation sources. For example, the natural exposure in the Ardennes region in southern Belgium is on average 2 mSv/year higher than in the Campine region in northern Belgium where SCK•CEN is located. The difference is due to the higher radon concentration and higher external exposure in the Ardennes. So the exposure to natural radiation sources in the Ardennes is over a period of 10 years about 20 mSv higher than in the Campine region, which is more than the average additional exposure received by the people living in the most contaminated areas around Chernobyl. The absence of aversion to live in the Ardennes has to do with the very different perception of natural versus accidental exposures.

### Reluctance of consumers to buy slightly contaminated food

Consumers have a strong aversion to buy food that could contain traces of contamination. This attitude has led to a decrease of the maximum food contamination levels in Europe and Japan in the aftermath of the Fukushima accident as shown in Table 2.

**Table 2.** Frequent changes of the European Union maximum food contamination levels for the sum of ^137^Cs and ^134^Cs after the Fukushima accident and the very low levels currently applicable in Japan.

	Infants foods (Bq/kg)	Milk (Bq/kg)	Other foodstuffs (Bq/kg)
EU: after Chernobyl	370	370	600
EU: after Fukushima (pre-established levels)	400	1000	1250
EU: since 13 April 2011 (old Japanese levels)	200	200	500
Japan: new levels since 1 April 2012	50^a^	50^a^	100

^a^The maximum contamination level with caesium of milk and food for infants in Japan is comparable to the concentration of natural ^40^K in milk of 45 Bq/l.

In the European Union, the maximum level for radioactive caesium in milk (sum of ^137^Cs and ^134^Cs) was first increased on 15 March 2011 from the post Chernobyl level of 370 Bq/l to the pre-established level of 1000 Bq/l and was decreased one month later, on 13 April 2011, by a factor of five to the Japanese level of 200 Bq/l. Complains from citizens about these changes resulted in an inquiry by the European Ombudsman^(9)^. On top of that, a year later, on 1 April 2012, Japan lowered its level by a factor of four to 50 Bq/l. And still the consumers in Japan continue to be very reluctant to buy local food.

### Most evacuees do not want to return to their old homes

In 1986, 115 000 people were forced to leave their homes in the 30 km exclusion zone after the Chernobyl accident. Thirty years later, only 400 elderly people, mostly poor farmers and former plant workers, have resettled inside the exclusion zone. At the same time, almost 7000 people still work at the Chernobyl power plant to decommission the site. Half of them live inside the exclusion zone for up to 14 days, while the others live on the borders of the exclusion zone and commute in.

A recent phenomenon is the emergence of tourism. The number of visitors of the Chernobyl accident site has increased to ~10 000 a year. The most popular site is the ghost town of Pripyat once home of 49 000 people, located just 3 km away from the reactor. Pripyat was built primarily to house workers from the Chernobyl nuclear power plant.

The government of Japan is determined not to get into a situation of a permanent exclusion zone and has engaged in a more than $10 billion effort to clean up the environmental contamination from the Fukushima accident. The intention is to reopen 70% of the evacuation zone to human habitation by 2017. Only the most contaminated areas near to the accident site would remain closed indefinitely.

The Japanese government lifted evacuation orders in two smaller areas previous to 5 September 2015, when the 7400 residents of the town of Nahara, one of municipalities within the 20 km radius that were evacuated in March 2011, were allowed to return home permanently. While some evacuees were determined to go back, many more did not want to return for various reasons. One of the main reasons is the fear of radiation, fuelled by the fact that it is not possible to clear away all the environmental contamination. There will always be some ^137^Cs contamination remaining with a half-life of 30 years. The government supplied radiation monitors and personal dosemeters to the residents to perform their own contamination measurements and to check their radiation levels in an attempt to become more familiar with the risk.

There are other reasons than fear of radiation why most of the evacuees do not want to go back.

- Most of the families with children have restarted lives elsewhere over the past few years.
- A lot of the younger evacuees have found a job elsewhere.
- Quite a few of the evacuees, particularly older people who have failed to find new livelihoods since the accident, are afraid to lose the compensation payments they need so badly.
- Farmers returning will probably not be allowed to grow the food crops they were familiar with for many years to come, due to the remaining ^137^Cs contamination.
- Lack of infrastructure and damage to houses in the towns abandoned since many years (almost no shops, doctors, schools, public transport available).

In the meantime many thousands of evacuees have joined lawsuits to demand more compensation so that they can choose for themselves whether to return, or to build new lives elsewhere.

## UNHELPFUL SCIENTIFIC CONTROVERSY ABOUT LOW-DOSE RISKS

The above examples illustrate that human behaviour is primarily driven by perceptions and not by facts. It also explains why a primarily scientifically driven approach with government measures expressed in µSv/h (dose rate), mSv/year (exposure), Bq/kg (food contamination) or kBq/m² (soil contamination) is not well accepted as it is not in line with the way the public perceives the risks from a nuclear accident. A government issuing only this kind of radiation protection measures will be faced with much opposition, fruitless discussions and conflicts of principle. On top of that comes the continuing controversy within the scientific community on the health implications of low doses of ionizing radiation, which results in conflicting messages to the population. On the one end of the scale, an alarming message based on the use of collective dose as an indicator of health risk and on the other end of the scale a reassuring message based on epidemiological considerations of no discernible increase in risk to be expected.

### Unhelpful approaches of Dealing with low-dose risks

The use of collective dose as an indicator of health risk at low individual doses sends an alarming message to the public. The concept is based on the translation of an individual risk with a low individual probability, to a collective risk with a theoretical number of victims. It fails to take into account our limited knowledge of the health risks at low doses of ionizing radiation and the fact that the ICRP system for operational radiation protection is based on practical but unproven assumptions, like the use of dose as a surrogate of risk and the extrapolation of the high-dose exposure risks to the low-dose range using the linear no-threshold (LNT) hypothesis. That is why the use of collective dose as a health risk indicator at low individual doses results in a simplistic and alarming message to the public.

Another approach at the other end of the scale is sending the misleading message from epidemiological studies of ‘no discernible increase in risk to be expected’, because it is based on the intrinsic limitations of epidemiological studies and not on scientific evidence of absence of health effects at low doses. As shown below, radiation epidemiology is at best a blunt instrument to determine health risks at low doses. Even the billion dollar study of the atomic bomb survivors lacks statistical significance below 150 mSv.

### Epidemiological studies lack statistical significance in the low-dose range

**Figure 1.**
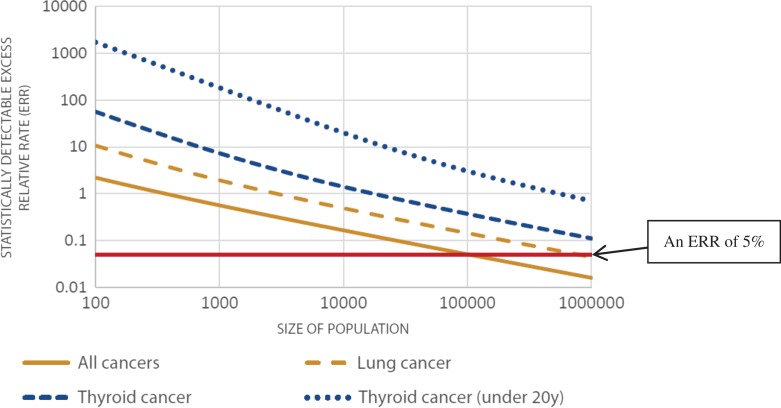
Size of cohort study needed to detect an ERR^(11)^.

The scientific community has relied heavily on the results of epidemiological investigations to quantify the increased frequency of occurrence of cancer in an exposed population. Figure XI of annex A of the UNSCEAR report of 2006^(10)^ shows the cancer mortality for different organs from the Life Span Study of the survivors of the atomic bombings in Japan. The excess relative risk (ERR) per unit dose for all cancers combined is 0.47 per Sv or a rounded value of 5% per 100 mSv assuming LNT. Figure 1 is adapted from figure A-II of the UNSCEAR report of 2012 on ‘Attributing health effects to ionizing radiation exposure and inferring risks’^ (11)^ and illustrates the minimum detectable ERR for ideal cohort studies.

Figure 1 shows that for all cancers combined two perfectly matched populations of 100 000 people are needed to detect an ERR of 5%, which corresponds to an exposure of 100 mSv in the study of the atomic bomb survivors. For specific cancers, like lung cancer or thyroid cancer, much larger cohorts are needed. The size of the cohort study is also likely to be larger due to bias and confounding factors^(11)^.

The average exposure in Belgium has doubled since the discovery of ionizing radiation in 1895, from ~2.3 to 4.6 mSv in 2006^(7–8)^. Of this increase 0.2 mSv comes from natural sources and 2.1 mSv from medical applications. During the same period the average life expectancy in Belgium has increased by 30 years, for men from 48 to 77 years and for women from 51 to 83 years. The combined effect of these two trends resulted in an increase of the average lifetime exposure by a factor of 3–4; for men from 110 mSv in 1895 to 354 mSv in 2006 and for women from 117 mSv in 1895 to 382 mSv in 2006. This high and increasing lifetime exposure effectively limits the power of low-dose epidemiological studies. There is, on top of that, a wide range of exposures. Quite a few people in the Ardennes, a radon prone area in Belgium, receive more than 10 mSv per year from natural sources (mainly from radon but also from gamma radiation) and quite a few patients receive more than 10 mSv per year from medical imaging in radiology and nuclear medicine. How would we be able to detect the health risks of a few tens of mSv with epidemiological studies if we already receive hundreds of mSv in our life from natural sources and medical imaging?

### Dosimetric limitations of low-dose radiobiology research

The absorbed energy from ionizing radiation is not uniformly distributed on the very small scale of the order of a cell nucleus, typically 5–10 µm. It is deposited close to the tracks with a density of ionization depending on the type and energy of the particle. The tracks of alpha particles (high-LET radiation) are two orders of magnitude more dense than the tracks of electrons (low-LET radiation). A dose of 10 mGy to a cell nucleus corresponds to about 10 tracks from electrons and only one track in every 25 cells from alpha particles^(12)^ (figure 2–1 on page 51 of the BEIR VI report). The natural background from low-LET radiation is of the order of 1 mGy/year or on average one track per cell nucleus per year. The intestinal epithelium of the alimentary tract system is a rapidly renewing tissue. A complete cellular replacement takes four to five days, which results in only one track from low-LET background radiation in every 80 cell nuclei, so that the vast majority of the intestinal epithelial cells will never be hit during their lifetime. These microdosimetric considerations on the frequency and density of tracks from ionizing radiation put a practical limit on low-dose radiobiology studies of a few mSv to a few tens of mSv.

### Radiobiology research and animal models can shed light on low-dose health effects

The difficulty to attribute specific cancer cases to low-dose exposure is mainly caused by

- The lack of a biomarker or a characteristic specific to radiation induced cancer.
- The long latency period between radiation exposure and cancer development. For instance, 45% of the cohort of atomic bomb survivors in Japan was still alive in December 2000.
- The high spontaneous cancer incidence in the general population not related to exposure to ionizing radiation. The lifetime risk of developing cancer in a well-developed country is of the order of 35–40%.

For diseases other than cancer, the same difficulties exist (hereditary effects, congenital malformations, cardio-vascular diseases, cataracts, etc.).

The affected population that has to live in a contaminated area and has to eat food that contains traces of contamination is not helped by the scientific controversy about low-dose risks nor by epidemiological studies in the low-dose range. What could shed light on low-dose health effects is basic research in radiobiology. The animal model in particular has an important role in understanding the effects of ionizing radiation on living organisms. Current molecular techniques are so sensitive that we can see all kinds of biological responses after very low doses of the order of a few mSv. We observe these biological effects on a daily basis in the lab (double strand breaks, activation and deactivation of gene networks, etc.). As these effects are transient in nature, their significance for the human health in the long term is still unclear given the absence of biomarkers specific to radiation exposure and the long latency period, which is for cancer in humans typically a few years to a few decades. Hence more radiobiology research in general and animal studies in particular is needed to bridge the gap between the transient short term effects and disease.

## HEALTH EFFECTS FROM IONIZING RADIATION VERSUS THE DISRUPTIVE IMPACT OF COUNTERMEASURES ON THE POPULATION

The Chernobyl accident caused acute radiation sickness amongst 134 rescue workers; 28 of whom died from this sickness in the first four months after the accident. Later, 19 more people of this group died of various diseases in the period 1987–2006^(6, 13)^. In contrast, no acute radiation sickness was diagnosed in rescue workers after the Fukushima accident^(14)^.

With regard to stochastic effects from the Chernobyl accident, there is clear epidemiological evidence of an increase of thyroid cancer amongst people who as children were heavily exposed to radioactive iodine. In the period 1991–2005, more than 6000 cases have been diagnosed and this higher risk is expected to remain for years^(13)^. Only a few people in whom thyroid cancer was diagnosed have passed away. Experience has shown that people suffering from this type of cancer have a very high chance of survival.

It is difficult to scientifically prove other health effects. This does not mean that there are no additional cancers or hereditary defects, but that we are not able to distinguish them from the normal occurrence of these diseases. A limiting factor for epidemiological studies is the bad economic situation in which the affected regions found themselves after the collapse of the Soviet Union, with a worsening of the health care and a decrease in the average life expectancy. Despite the difficult situation, there is some epidemiological evidence of an increase of leukaemia and the development of cataract among recovery workers. The Chernobyl Forum, a collaboration between eight agencies of the United Nations and the governments of Ukraine, Belarus and Russia, has made in 2005 an estimate of the number of additional deaths in the highest exposed population groups. The total number of deaths attributable or to be expected in the future was estimated at 4000^(15)^.

Latency periods of cancer range from several years to decades, so that for most of the cancer types it is still too early to develop cancer from the Fukushima accident. In addition, the cancer incidence to be expected from the doses received by the workers and the local population will probably be too low to distinguish them from the normal occurrence of these cancers^(14)^.

In the Chernobyl and Fukushima accidents, the government had to take very disruptive measures to protect the population from high exposures to ionizing radiation. In both cases, more than hundred thousand people close the accident site were evacuated within a few days. People further away had to take shelter in their homes for a period of time and the interdiction of contaminated foods affected populations at great distances from the accident site. These countermeasures deeply disturbed the life of the local population, resulting in various stress-related health complains, including depression and post-traumatic stress disorder.

## ENGAGE IN DIALOGUE TO CHANGE THE NEGATIVE PERCEPTION

The examples developed earlier, namely the aversion of having to live in a contaminated territory, the reluctance to buy even slightly contaminated food and the opposition of most evacuees to return to their old homes, illustrate the great uneasiness of the affected populations with a purely technical and science-based governmental approach. Implementing a policy from a top-down perspective ignoring the social dimension conflicts with the values and beliefs of the public with respect to the various consequences of a nuclear accident and its health implications. Particular unhelpful in the communication to the public are the conflicting messages from the scientific controversy about low-dose risks and from epidemiological studies in the low-dose range. What could shed light on the low-dose health effects is more basic research in radiobiology, but this is a long-term goal.

In order to overcome these problems it is important to pursue a bottom-up approach by involving civil society and local actors. Both approaches (top-down and bottom-up) should be combined as the social and technical aspects of the decision and implementation of countermeasures in the aftermath of a nuclear accident are intertwined and subject to change over time. Engaging in direct dialogue with the affected population and granting them a say in the decision-making processes will give the feeling of having some control over the situation. The practical implementation requires, once the acute phase of the accident is over, the creation of a formal consultation structure, of local bodies and committees (a kind of partnership) funded by the authorities but operated by the local community. The partnership should be given sufficient financial means to consult experts on their own, to perform radiation measurements and to put radiation monitors at the disposal of the population concerned. Such a participatory approach is very demanding for the authorities, but is likely to have a positive influence on the negative perception of the affected populations and can accomplish a change of their state of mind from ‘victims to survivors’.
